# Establishment of a Pre-vascularized 3D Lung Cancer Model in Fibrin Gel—Influence of Hypoxia and Cancer-Specific Therapeutics

**DOI:** 10.3389/fbioe.2021.761846

**Published:** 2021-10-14

**Authors:** Caroline Kniebs, Anja Elisabeth Luengen, Daniel Guenther, Christian Gabriel Cornelissen, Thomas Schmitz-Rode, Stefan Jockenhoevel, Anja Lena Thiebes

**Affiliations:** ^1^ Department of Biohybrid and Medical Textiles (BioTex), AME - Institute of Applied Medical Engineering, Helmholtz Institute, RWTH Aachen University, Aachen, Germany; ^2^ Aachen-Maastricht Institute for Biobased Materials, Faculty of Science and Engineering, Maastricht University, Brightlands Chemelot Campus, Geleen, Netherlands; ^3^ Clinic for Pneumology and Internal Intensive Care Medicine (Medical Clinic V), RWTH Aachen University Hospital, Aachen, Germany

**Keywords:** 3D *in vitro* cancer models, drug testing, fibrin gels, tumor model, lung cancer

## Abstract

Lung cancer is the most frequently diagnosed cancer worldwide and the one that causes the highest mortality. In order to understand the disease and to develop new treatments, *in vitro* human lung cancer model systems which imitate the physiological conditions is of high significance. In this study, a human 3D lung cancer model was established that features the organization of a tumor with focus on tumor angiogenesis. Vascular networks were formed by co-culture of human umbilical vein endothelial cells and adipose tissue-derived mesenchymal stem cells (ASC) for 14 days in fibrin. A part of the pre-vascularized fibrin gel was replaced by fibrin gel containing lung cancer cells (A549) to form tri-cultures. This 3D cancer model system was cultured under different culture conditions and its behaviour after treatment with different concentrations of tumor-specific therapeutics was evaluated. The evaluation was performed by measurement of metabolic activity, viability, quantification of two-photon laser scanning microscopy and measurement of the proangiogenic factor vascular endothelial growth factor in the supernatant. Hypoxic conditions promoted vascularization compared to normoxic cultured controls in co- and tri-cultures as shown by significantly increased vascular structures, longer structures with a higher area and volume, and secretion of vascular endothelial growth factor. Cancer cells also promoted vascularization*.* Treatment with 50 µM gefitinib or 50 nM paclitaxel decreased the vascularization significantly. VEGF secretion was only reduced after treatment with gefitinib, while in contrast secretion remained constant during medication with paclitaxel. The findings suggest that the herein described 3D lung cancer model provides a novel platform to investigate the angiogenic potential of cancer cells and its responses to therapeutics. Thus, it can serve as a promising approach for the development and patient-specific pre-selection of anticancer treatment.

## Introduction

Lung cancer is the most common type of cancer in the word and has the highest lethality. Every year, 1.8 million lung cancer patients are diagnosed and 1.6 million people die of lung cancer worldwide. Lung cancer (= bronchial carcinoma) consists of four subtypes, the malignant forms of the cancer. The most common are non-small cell lung carcinomas (NSCLC); they account for approximately 75–80% of all lung carcinomas. Most commonly, patients are surgically treated (Doss et al., 2007). The aim is the complete removal of the tumor and a successful long-term cure. This is usually only possible in the early stages of the disease, but lung carcinomas are characterised by rapid growth as well as early metastasis (Kalemkerian, 2016; Blandin Knight et al., 2017). Consequently, the disease is often already in an advanced stage at diagnosis and has a poor prognosis. For advanced and metastatic stages, other therapies are available, such as chemotherapy, radiotherapy or radiochemotherapy. Newer approaches such as targeted, immunotherapeutic treatments or both may also be considered, however, also with poor prospects of success, as these are highly complex at the molecular level due to a high mutation rate (Peifer et al., 2012; Elliott et al., 2020).

To improve treatment options, it is necessary to gain a better understanding of the molecular mechanisms of the tumor angiogenesis as well as to identify carcinogens and to develop personalized treatments and drugs (Katt et al., 2016; Liaw et al., 2018). The tumor mircroenvironment plays a crucial role in this process: the cancer cells are supplied by diffusion of nutrients and oxygen from the surrounding tissue. During further growth of the tumor, cells located centrally in the tumor tissue are no longer sufficiently supplied with nutrients and oxygen due to the greater distance to the surrounding blood vessels. As a result, the cells undergo death by apoptosis or necrosis, limiting further growth of the tumor (Baeriswyl and Christofori, 2009). The resulting balance between proliferation and death of cancer cells is referred to as angiogenic dormancy (Endo and Inoue, 2019). Tumor angiogenesis is the resulting transition to the vascular stage of the tumor growth and is triggered by stimuli such as hypoxia and the release of different pro-angiogenic cytokines like vascular endothelial growth factor (VEGF) (Carmeliet and Jain, 2011).

Animal models and 2D *in vitro* models have been investigated in order to improve the comprehension of the molecular mechanisms of tumor angiogenesis, but both have shortcomings in mimicking the behaviour of human tumors *in vitro* (Barré-Sinoussi and Montagutelli, 2015). While the challenge in 2D *in vitro* models is to mimic the microenvironment of the degenerated cells, animal models lack a full transferability to humans (Malyla et al., 2020). In lung cancer research, mouse models are primarily used to study pathogenesis. Although the human genome and the mouse genome are closely aligned, they strongly differ with respect to the physiology of the lung, therefore the translation of experimental data to humans is often not feasible (Mural et al., 2002; Miller and Spence, 2017). 3D *in vitro* models represent an alternative to animal experiments and have the advantage, that the transfer to humans can be ensured by using cells of human origin, especially with regard to drug responses (Doke and Dhawale, 2015).

In published studies with multicellular 3D cancer models, which represent the interaction between cancer cells and non-malignant cells, different relevant cell types were chosen, but the tumor microenvironment was represented in a simplified way regarding tumor cell migration and the surrounding vascular network (Li et al., 2018; Dong et al., 2019). The importance of cell-cell contacts and vascularization in a tumor drug model has been demonstrated in acute myeloid leukemia models, in which 3D tri-culture models based on hydrogels (endothelial cells, pericytes and cancer cells) had an increased resistance to tumor drugs compared to 2D and 3D mono-tumor-cultures (Koch et al., 2020).

It was already shown in previous studies, that the formation of vascularization is induced *in vitro* by endothelial cells in co-culture with supporting cells such as fibroblasts and mesenchymal stem cells in different scaffolds. Thereby, a branched elongated network with lumen formation within the same range as human capillaries could be observed (Kniebs et al., 2019; Kreimendahl et al., 2019).

The aim of this study is to mimic the natural tumor mircroenvironment in hydrogels. For this purpose, a 3D lung cancer model in fibrin hydrogels with tri-cultures is developed, which is based on the natural structure of the tumor mircroenvironment. In particular, the influence of cancer cells on an existing vascular network and the associated tumor angiogenesis will be illustrated. The influence of culture time and conditions are examined and defined concentrations of the tumor drug gefitinib and paclitaxel are tested on the model. Samples are analysed and quantified by two-photon laser scanning microscopy (TPLSM).

## Materials and Methods

### Isolation and Cell Culture

The local ethics committee of the Medical Faculty of RWTH Aachen University (EK 197/19) approved the isolation and application of primary human cells. Briefly, human umbilical vein endothelial cells (HUVECs) were isolated from umbilical cords provided by the Clinic for Gynaecology and Obstetrics (RWTH Aachen University Hospital) and cultured in endothelial cell growth medium (EGM2, PanBiotech) up to passage 4. Adipose derived mesenchymal stem cells (ASCs) were isolated from abdominal fat tissue provided by the Clinic for Plastic, Hand and Burns Surgery (RWTH Aachen University Hospital) and cultured in Mesenpan medium (PAN-Biotec) with 2% fetal calf serum (FCS, Thermo Fisher Scientific) and 1% antibiotic/antimycotic solution (ABM, Gibco) up to passage 5. The confirmation of HUVECs and ASCs was shown in previous studies using the same isolation protocol (Kniebs et al., 2019; Kreimendahl et al., 2019). Furthermore, the MSCs isolation is regularly verified by flow cytometry according to Dominici et al., 2006 (Dominici et al., 2006). ASCs were positive for CD90, CD73 and CD105 with ≥97.2%. The markers CD45, CD34, CD11b, CD79alpha and HLA-DR surface molecules were negative (≥98.7%). The tumor cell line A549 was purchased (DSMZ-German Collection of Microorganisms and Cell Cultures GmbH) and expanded with Dulbecco’s Modified Eagle’s medium (DMEM, Gibco) with 10% FCS and 1% ABM.

### Hydrogel Moulding Process and Culture

**FIGURE 1:**
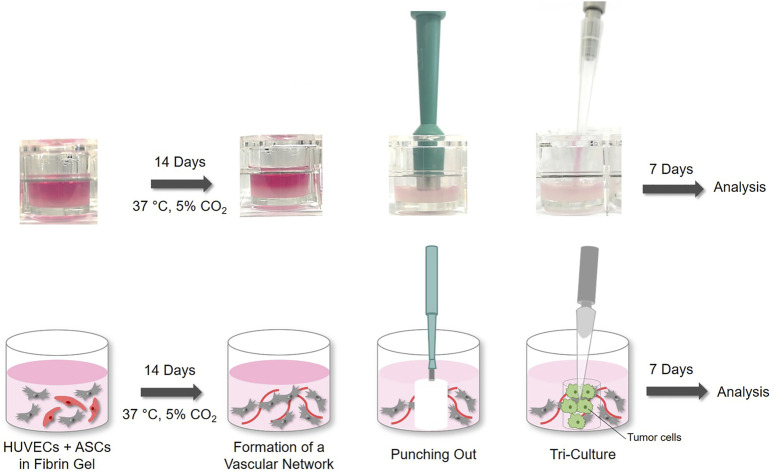
Experimental Set-up: HUVECs and ASCs were moulded in a fibrin gel and cultured for 14 days at 37°C and 5% CO_2_. To form tri-cultures, the central part of the gel was punched out and replaced by fibrin gel containing A549 cancer cells.

HUVECs and ASCs were suspended with a final concentration of 3.0 × 10^6^ cells/ml fibrin gel in a final volume of 350 µl. The fibrin hydrogels were moulded according to established protocols (Kniebs et al., 2019). Briefly, fibrin hydrogels were moulded with final concentrations of 5 mg/ml fibrinogen (VWR), 3 U/ml thrombin (Sigma-Aldrich) and 3.75 mM CaCl_2_ and the corresponding cell suspension into 24 well plates. The polymerization was enhanced by incubating the samples for 30 min at 37°C and 5% CO_2_. The hydrogels were incubated at 37°C, 5% CO_2_ and 21% O_2_ for normoxic culture conditions and at 5% O_2_ for hypoxic culture condition, respectively. The samples were cultured in EGM-2 medium with 0.16 mg/ml tranexamic acid (Cyclokapron, Pfizer), medium change was performed every second day. After 14 days, in order to form the tri-cultures, a central part was punched out of the fibrin gels with a 4 mm biopsy punch (pfm medical) and replaced by a 100 µl fibrin gel with cancer cells with a final concentration of 3.0 × 10^6^ cells/ml hydrogel (Figure 1).

Gefitinib (G-4408, LC Laboratories) was solved with a concentration of 100 mg/ml in DMSO and subsequently diluted in the respective concentrations in EGM-2 medium. Paclitaxel (Merck, T7402-5 MG) was solved in 50 mg/ml DMSO and diluted into the respective concentrations in EGM-2 medium.

The samples were treated for 24 and 72 h with the different drug concentrations at 37°C and 5% CO_2_. All experiments were carried out using cells from three different donors and three technical replicates.

### Metabolic Activity

The Cell Proliferation Kit II (XTT, Merck) was used to quantify cell proliferation and metabolic activity with different drug concentrations to determine the mean inhibitory concentration (IC_50_ values) of 2D and 3D tri-cultures. The assay was carried out according to DIN EN ISO 10993-5 and measured after 24 and 72 h incubation time. For the 2 D cell culture, the cells of the tri-culture were seeded in a concentration of 1.0 × 10^6^ cells/ml per cell type and exposed to the different drug concentrations of the medium for 72 h. The hydrogels for the 3D cell culture were moulded as described above.

### AlamarBlue™ Assay

The alamarBlue™ assay is used for the quantitative determination of cell viability, proliferation and cytotoxicity (Präbst et al., 2017). The alamarBlue™ reagent was initially diluted 1:10 in EGM-2 medium (alamarBlue™ measuring reagent I) on the measurement days. After removal of the culture medium, 300 µl of the measurement reagent was added to the fibrin gels. The mixtures were incubated for 2 h at 37°C in the dark. Subsequently, 100 µl of the supernatant was pipetted in a 96-well plate and the fluorescence was measured with an excitation wavelength of 560 nm and an emission wavelength of 590 nm.

### ELISA

The influence of the pro-angiogenic cytokine VEGF onto the different culture condition was determined by a sandwich enzyme-linked immunosorbent assay (ELISA) using a multiplex ELISA kit (VEGF Human ELISA Kit, ThermoFisher Scientific) according to manufacturer´s instructions. Statistical significance was determined using the GraphPad Prism software 9.0. For this purpose, a two-way ANOVA and Tukey’s post hoc significance test were performed. The significance level was set at *p* < 0.05 (*).

### 2-Photonmicroscopy and Quantitative Analysis

The samples were stained and analysed according to established protocols (Kniebs et al., 2019; Kreimendahl et al., 2019). The cells were stained with the fluorescent Vybrant™ dyes DiO, Dil and DiD (ThermoFisher Scientific) according to manufacturer´s instructions. Additionally, the samples to determine the influence of tumor-specific therapeutics were stained with mouse anti-CD31 (PECAM-1, 1:100; Sigma-Aldrich) and Alexa Fluor^®^ 594 goat anti-mouse IgG (1:400, Acris Antibodies). The quantitative evaluation was performed with a two-photon laser scanning microscope, images processed and evaluated with Imaris 9.6.0 software (Bitplane Inc. South Winsor). The vascular structures were quantified according to their average volume, surface area, length, total number of vascular structures and branching points.

### Statistical Analysis

Statistical significance was determined using the GraphPad Prism software 9.2.0. After confirming that the assumptions of normality and equal variance were met, a two-way ANOVA and Tukey’s post hoc significance test were performed. The values that were not normally distributed were analysed using Friedman test and Dunn´s multiple comparisons. The significance level was set at *p* < 0.05 (*).

## Results

### The 3D Lung Cancer Model

**FIGURE 2:**
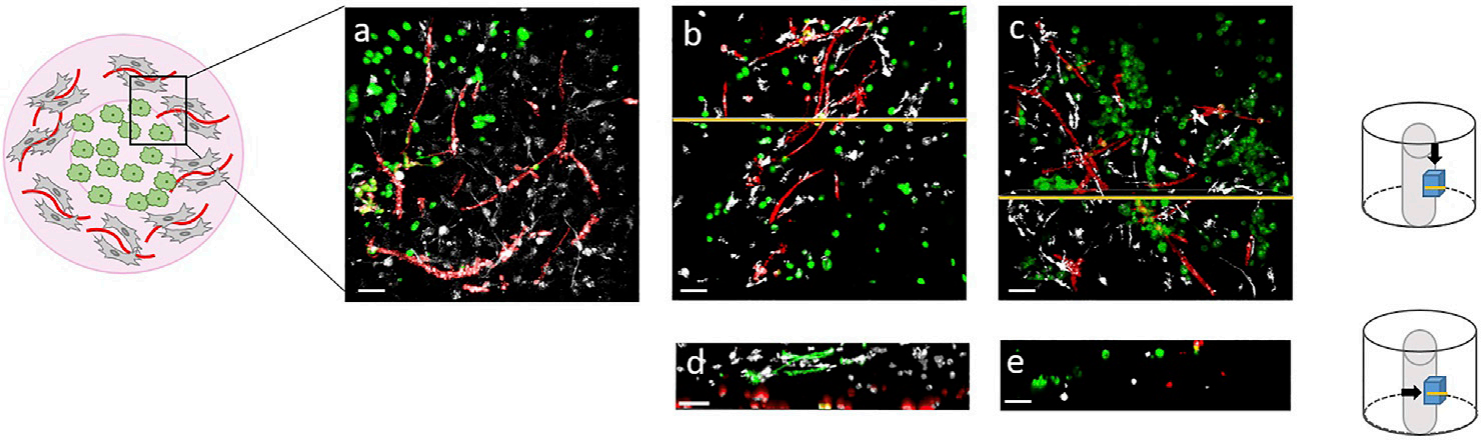
Tri-cultures of HUVECs (red), ASCs (grey) and A549 lung cancer cells (green) in fibrin gels. Top view **(A–C)** and cross-sections **(D–E)** of the gels after 21 days of culture (TPLSM images). Scale bar: 50 µm.

To mimic tumor angiogenesis *in vitro*, a 3D lung cancer model was established with tri-cultures of HUVECs, ASCs and A549 lung cancer cells in fibrin gel. For this purpose, a fibrin gel was pre-vascularized with HUVECs and ASCs and after 14 days, the central part of the hydrogel was then replaced by A549 cells embedded in fibrin gel. The vascularization of the gels was investigated after a total of 21 days of culture. HUVECs (red) and ASCs (grey) build capillary-like vessel structures throughout the outer part of the hydrogel (Figure 2A). Highly branched and elongated structures (red) were observed in all cultures with all three donors. The vascular structures are in close proximity to the cancer cells. However, as can be seen in the cross-sections, no direct interaction between cancer cells and vascular structures could be detected (Figure 2B,C).

### Influence of Oxygen Level and Cancer Cells on Vascularization Parameters

**FIGURE 3:**
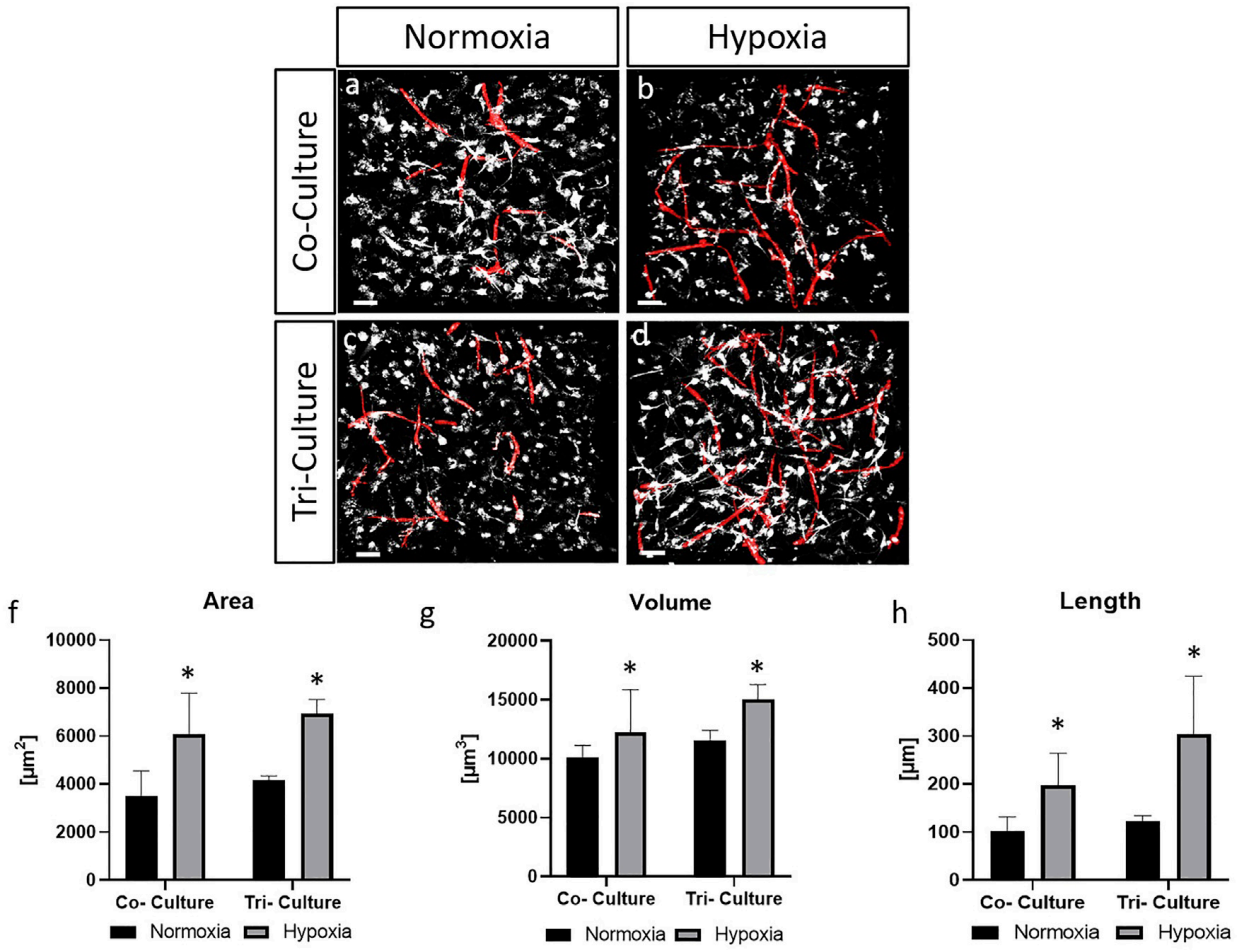
CD31-stained HUVECs (red) and ASCs (grey) in co- and tri-cultures under normoxic and hypoxic culture conditions **(A-D)**, TPLSM images. Scale bar: 50 µm. Statistical evaluation of the parameters: area of the vascular structures **(F)**, volume **(G)** and total length of the structures **(H)**. The statistical significance is provided with * and shows the significance of the individual values for *p* < 0.05 and corresponding to the same cultures but different culture conditions.

Co-cultures (HUVECs and ASCs) and tri-cultures were cultured for 3 days (day 15–18) under hypoxic culture conditions (5% O_2_) or continuous normoxic conditions (21% O_2_) during the 21-days culture time and were evaluated in terms of vascularization parameters in the outer parts of the hydrogels. Depending on the culture condition, varying degrees of vascular structures were formed (Figure 3). Co-cultures under hypoxic conditions showed increased formation of vascular structures compared to co-cultures after 21 days of normoxic culture (Figure 3A,B). Similar results were observed in the tri-cultures. The results are confirmed by the quantitative evaluation (Figure 3C,D).

Significant differences were revealed in the co- and tri-cultures between normoxic and hypoxic culture for the parameters area (Figure 3F), volume (Figure 3G) and length (Figure 3H). Under hypoxic conditions, the structures detected in co-cultures were twice as long than under normoxic conditions, 101 ± 30 µm vs 197 ± 66 μm, and almost three times longer, 123 ± 10 µm vs 304 ± 120 μm, in tri-cultures. The values for the parameters area, volume and length were significantly higher in co- and tri-cultures under hypoxic culture conditions. Furthermore, the vascularization in tri-cultures was higher in for all parameters and in all samples than in the co-cultures, but not significantly. For analysis of the influence of hypoxic culture and cancer cells on the secretion and consumption of the proangiogenic cytokine VEGF, an ELISA was performed. This was carried out with media supernatants from hypoxic and normoxic cultured co- and tri-cultures, which were collected after 21 days of culture. The measured values are shown in relation to the corresponding measured values, meaning the VEGF concentration of fresh medium was respectively subtracted, such that positive values correspond to secretion and negative values to consumption of the cytokine VEGF.

**FIGURE 4:**
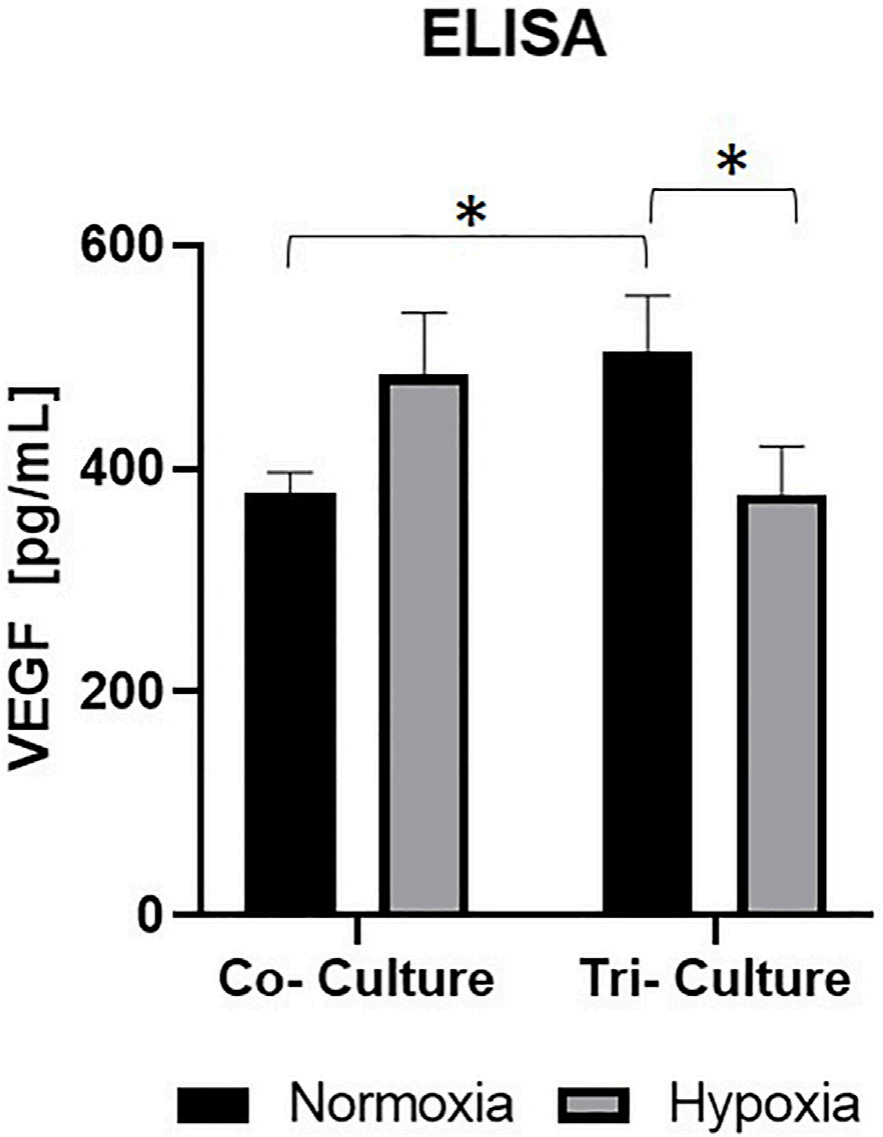
ELISA of co-and tri cultures under different culture conditions with the pro-angiogenic cytokine VEGF. VEGF concentration in cell-free medium was subtracted from all measured values. Error bars show standard deviation. Statistical significance indicated by * shows significance (*p* < 0.05) between mean values.

Under normoxic conditions, tri-cultures secreted more VEGF than co-cultures. Under hypoxic conditions, the opposite was observed, with co-cultures secreting more VEGF than tri-cultures. An increase in VEGF secretion was observed after hypoxic culture of co-cultures, 484 ± 55 μm, in comparison to normoxic cultivation, 379 ± 17 µm. In contrast, in tri-cultures more VEGF was produced under normoxia, 505 ± 51 μm, than under hypoxic culture conditions, 376 ± 44 µm (Figure 4).

For further investigations of the model, the samples were cultured for 21 days with 3 days of hypoxic culture conditions.

### Influence of Cancer-specific Therapeutics on Cells and Vascularization

**FIGURE 5:**
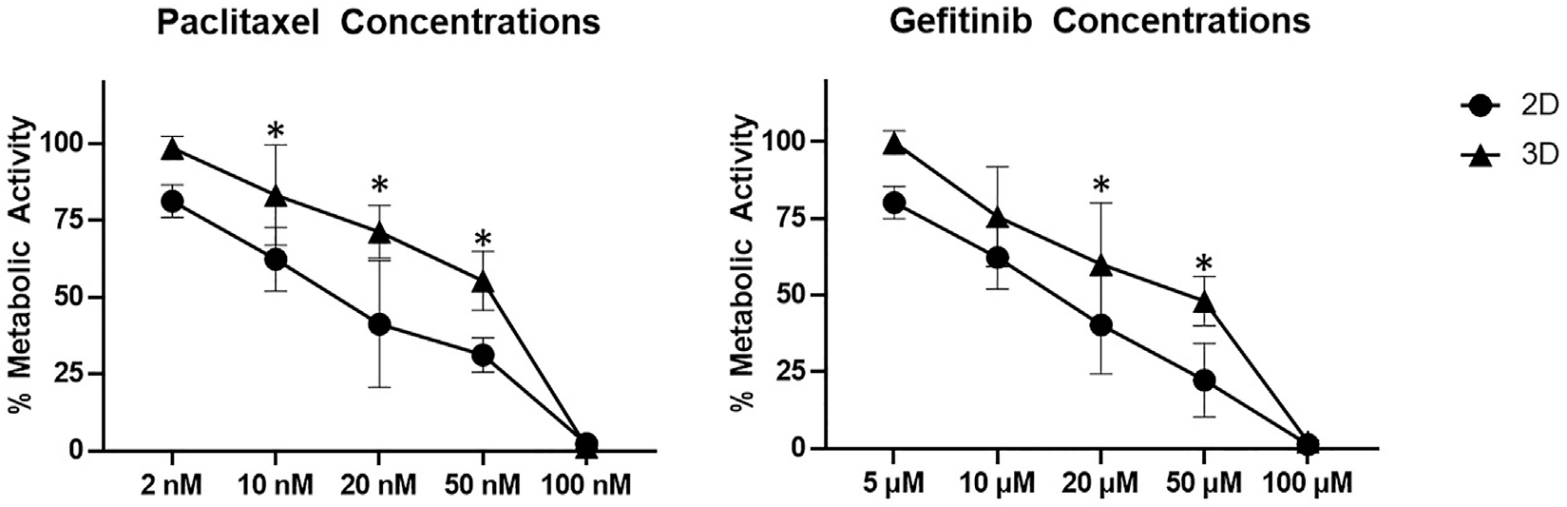
Meatbolic activity measured by an XTT-assay after treatment of 2D and 3D Tri-cultures with different concentrations of gefitinib and paclitaxel for 72 h. Error bars show standard deviation. Statistical significance indicated by * shows significance (*p* < 0.05) between mean values.

In order to investigate the functionality of the cancer model and the vascularized network, 2D and the 3D tri-cultures were treated with five different concentrations of the tumor-specific therapeutics gefitinib and paclitaxel for 72 h. The metabolic activity was measured with an XTT assay and is displayed in relation to the negative control, to compare the effective concentrations for 2D and 3D cultures (Figure 5). Tri-cultures in 2D were more sensitive to the drug doses applied in both trials. Tri-cultures in 3D were significantly more resistant when treated with 10 μM, 20 and 50 µM gefitinib or 10 and 20 nM paclitaxel.

**FIGURE 6:**
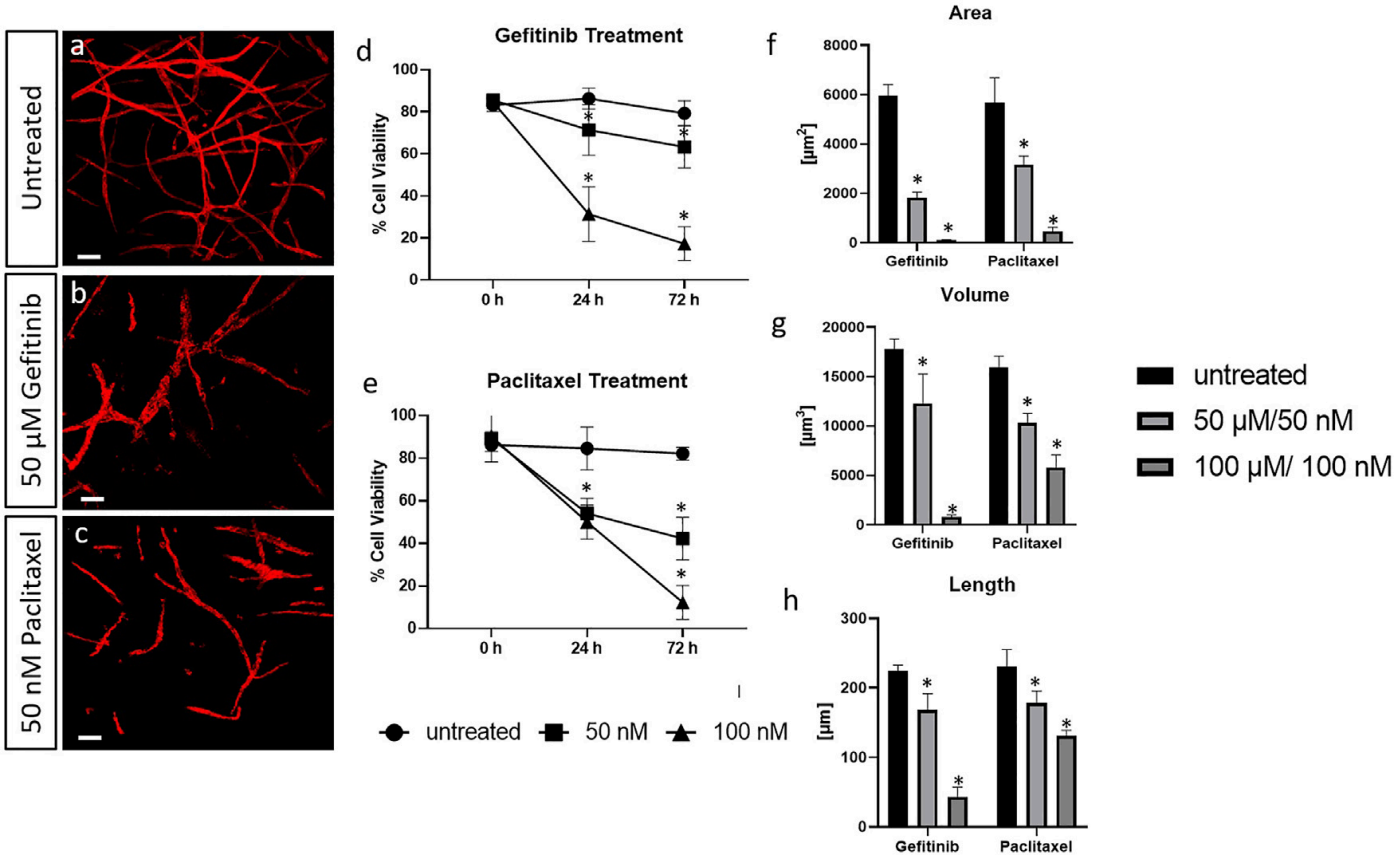
CD31-stained HUVECs (red) in tri-cultures under hypoxic culture conditions **(A)** after treatment with 50 µM gefitinib and 50 nM paclitaxel for 72 h **(B)** and **(C)**, respectively; TPLSM images). Scale bar: 50 µm. Cell survival assay of tri-cultures in 3D after treatment with different doses of gefitinib and paclitaxel **(D, E)**. Statistical evaluation of the parameters: area of the vascular structures **(F)**, volume **(G)** and total length of the structures **(H)**. Statistical significance is provided with * and shows the significance of the individual values for *p* < 0.05.

The half-maximal inhibitory concentrations, 50 µM gefitinib and 50 nM paclitaxel in 3D, were then applied in the 3D cancer models after 21 days of culture and analysed after 72 h. TPLSM revealed a wide spread vascularized network in the control without drug treatment (Figure 6A). A decreased vascularization was observed after treatment with 50 µM gefitinib (Figure 6B) and 50 nM paclitaxel (Figure 6C). Additionally, the cell viability decreased to 43–62% after 72 h of drug treatment with the respective concentrations as measured by an AlamarBlue-assay. Treatment with 100 µM gefitinib and 100 nM paclitaxel resulted in a decrease of the viability to approximately 5% (Figure 6D-E).

The obtained results are supported by the quantification of the vascularized structures. After 72 h of treatment with 50 µM gefitinib, the vascularization of the tri-cultures was significantly reduced in all three donors displayed by a reduction in the area, volume and length of the vascularized structures. The treatment with 100 µM gefitinib resulted in an absence of elongated structures (Figures 6 F,G). Similar results were obtained for the treatment with paclitaxel. With 50 nM, a significant reduction of approximately 50% was detected for structure area and volume (Figures 6 F,G). The qualitative results of the TPLSM evaluation of both drug treatments are supported by the quantitative evaluation of the different structure parameters and cell viability.

**FIGURE 7:**
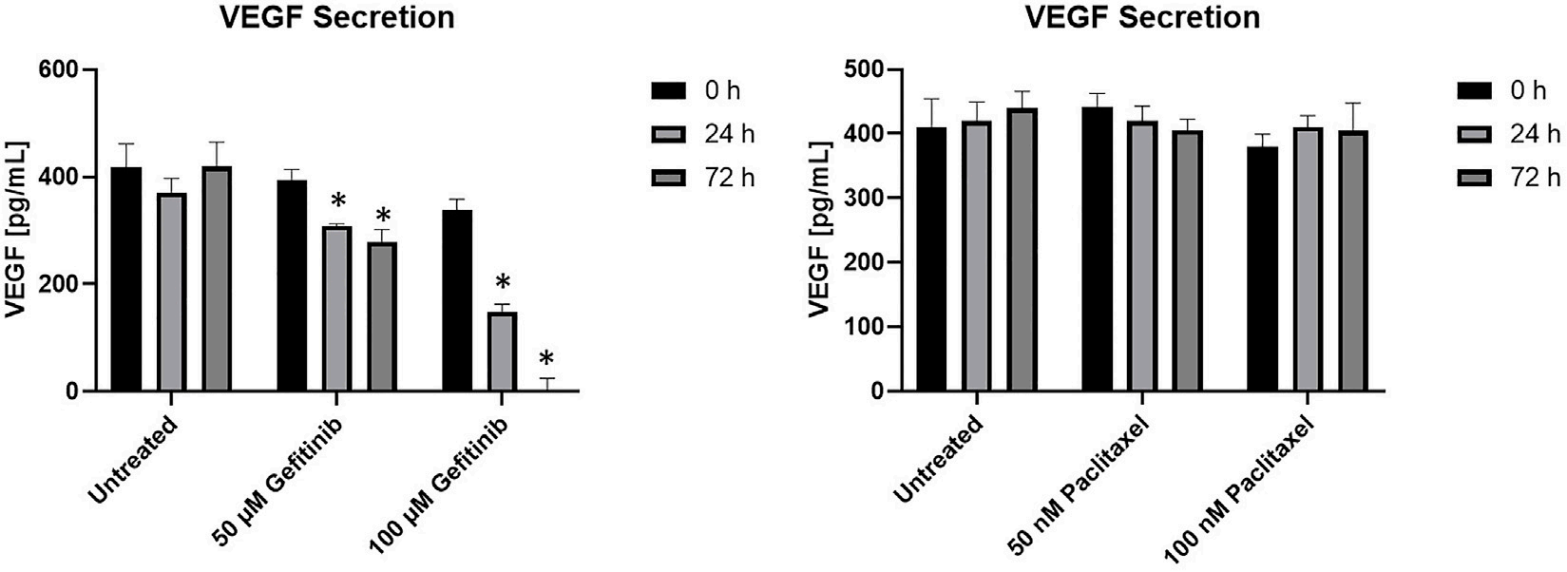
ELISA of tri-cultures with different gefitinib **(A)** and paclitaxel concentrations **(B)** at different time points with the pro-angiogenic cytokine VEGF. VEGF concentration in cell-free medium was subtracted from all measured values. Error bars show standard deviation. Statistical significance indicated by * shows significance (*p* < 0.05) between mean values.

For analysis of the influence of the drug concentration on the secretion and consumption of the proangiogenic cytokine VEGF, an ELISA was performed (Figure 7). Hereby, the measured values are shown in relation to the corresponding measured values, meaning the VEGF concentration of fresh medium was respectively subtracted.

The normalized VEGF secretion of tri-cultures treated with gefitinib decreased significantly compared to the increased dosage of the tumor specific therapeutic after 24 and 72 h (Figure 7A). The treatment with the half and maximal inhibitory concentration of paclitaxel had no impact on VEGF secretion in the tri-cultures. Here, the VEGF in the supernatant remained stable at around 400 pg/ml (Figure 7B). Additionally, in 2D cell viability and metabolic activity assays of A549 cells with different paclitaxel concentrations, no reduction in viability could be observe (see Supplementary Data).

## Discussion

Diseases of the respiratory system, including lung cancer, are one of the major causes of mortality and morbidity worldwide (Robert Mason et al., 2010). *In-vitro* models that encompass the complexity of the respiratory system are required for a better understanding of cancer pathological processes and foster the development of new treatments (Sedláková et al., 2019).

3D cell culture enables the development of physiologically relevant cancer models based on human cells that allow new insights into the mechanisms of the disease and can contribute to the development of new therapies (Nath and Devi, 2016). Furthermore, by using patient cells, 3D models can provide insight into effective, personalised therapy options (Fong et al., 2017). However, it is essential to establish *in vitro* models that combine vascularized stromal and parenchymal compartments to mimic the natural microenvironment with cell-cell contacts and secretion of angiogenesis related cytokines (Teixeira et al., 2021). Amann *et al.* developed a multicellular 3D cancer model made by hanging drop technology, in which they could observe the tumor mircroenvironment and aggregation of the different cell types, but without *in vitro* vascularization of the model (Amann et al., 2017). Even though some studies aimed to combine *in vitro* vascularization and tumor mircroenvironment, to the authors’ knowledge, there is none reported with a pre-vascularized network with lung cancer cells cultured under different conditions and in combination with treatment with tumor-specific therapeutics (Song et al., 2014; Amann et al., 2017; Koch et al., 2020).

To overcome these limitations, we developed a pre-vascularized 3D lung cancer model: a tri-culture hydrogel model consisting of HUVECs, ASCs and cancer cells. The benefits of using fibrin as a hydrogel are the high biocompatibility that it possesses as a natural polymer, as well as the mechanical properties that enable the complex representation of the tumor-tumor mircroenvironment interaction (Rodrigues et al., 2021). In addition, the co-culture of HUVECs and supporting cells such as ASCs or fibroblasts in fibrin gels for the formation of vascular structures was already successfully established (Kniebs et al., 2019; Kreimendahl et al., 2019). Here, the influence of cancer cells on the vascularization was investigated after 21 days. The cultivation period has been chosen as optimal time point of vascularization in fibrin gels and therefore in this study at time point of adding the tumor cells to the pre-vascularised network to obtain the mature tumor model after 21 days. All three HUVECs donor experiments showed high vascularization in tri-cultures with cancer cells and all evaluated structural parameters assessed were increased compared to the control group. Cancer cells can indirectly influence vascularization through paracrine secretion of proangiogenic factors (Nath and Devi, 2016). For example, Ishibashi *et al.* showed that conditioned medium taken from the supernatant of a tumor cell culture stimulates angiogenesis of endothelial cells (Ishibashi et al., 1995). Here, the angiogenesis stimulation of cancer cells was induced by basic fibroblast growth factors (bFGF) expression. bFGF binding proteins are secreted from cancer cells into the ECM, and bind to bFGF which activated and stimulated angiogenesis in combination with VEGF (Ishibashi et al., 1995).

A limitation of our model is the lack of migration of the tumor into the pre-vascularised part of the hydrogel. Cancer cells migrate towards the border of the gel and thus into the immediate proximity of the co-culture, but no direct interaction was observed. This is presumably because no connection between the gels is formed after moulding fibrin gel with cancer cells into the pre-vascularized fibrin gel. As a future perspective to overcome this limitation, the direct interaction between already formed vascular structures and cancer cells could potentially be investigated by using fibrin gels with varying pore sizes, to achieve a simplified migration of the cells. Teixeira *et al.* have implemented porous alginate scaffolds produced by freeze-drying for their tri-culture hybrid model with breast cancer cells. However, the *in vitro* vascularization found in this model was not as extended as in our model (Teixeira et al., 2021). The future model has to balance sufficient elasticity for *in vitro* vascularization and adequate porosity for cell migration and overcome the limitation of the two-phase hydrogel.

In a second experiment, the influence of oxygen concentration on vascularization was investigated. Co- and tri-cultures showed a higher vascularized network under hypoxic culture conditions with significantly higher parameters for area, volume and length. Furthermore, the ELISA showed an increased secretion of VEGF in co-cultures after hypoxic culture compared to normoxic culture. Hypoxia is the most important mediator of physiological angiogenesis. When the oxygen level drops below a threshold, the cancer cells become hypoxic and start secreting pro-angiogenic factors like VEGF (Clapp et al., 2009; Heinolainen et al., 2017). Briefly, hypoxia initiates a signalling cascade of hypoxia-inducible factor-1 (HIF-1) and VEGF-mediated pathways that induce tumor vascularization by promoting sprouting and outgrow process of blood vessels (Jiang et al., 1996). The amount of HIF-1α activity in the nucleus fluctuates in correlation with oxygen levels in cells. This HIF activation also upregulates major angiogenesis-regulating signalling molecules such as VEGF-A and its receptors VEGFR-1 and VEGFR-2, which are involved in vascularization (Wenger, 2002). Additionally, the results are consistent with previous studies, where HUVECs showed an enhanced proliferation rate when cultured under 5% O_2_ and co-cultures with HUVECs and stem cells even showed enhanced formation of vessel-like structures (Ishibashi et al., 1995; Yuan et al., 2015). However, the ELISA results from the tri-cultures showed a higher VEGF secretion in normoxic cultured samples than in hypoxic ones while vascularization was increased nevertheless. This could be due to the fact that angiogenesis of surrounding blood capillaries is induced *in vivo* only as a consequence of tumor cell adaptation to oxygen and nutrient deprivation by secretion of proangiogenic factors (Ribatti et al., 2007; Baeriswyl and Christofori, 2009). Consequently, cancer cells could react with a delay to hypoxic culture with increased secretion of proangiogenic cytokines, which is why an increase in VEGF secretion could not be detected at the time of hypoxic culture.

The final model with a total culture time of 21 days including 3 days of hypoxic culture was further investigated towards its functionality regarding the influence of tumor-specific therapeutics. Gefitinib, an epidermal growth factor receptor inhibitor and licensed under the trade name Iressa, has been used in the clinic since 2009 to treat patients with non-small cell lung cancer (Dhillon, 2015). Paclitaxel, which induces an interferences of the normal function of microtubules during cell division, has been used in clinic against advanced non-small cell bronchial carcinoma (Ramalingam and Belani, 2004). In our study, 2D and 3D tri-cultures were exposed to different concentrations of the two drugs for 72 h to measure the cells’ metabolic activity. Tri-cultures in 2D were more sensitive to the drug doses applied in both trials than the cultures in 3D. Other studies support that cells cultured in 2D respond more sensitively to drugs than those cultured in 3D (Imamura et al., 2015; Koch et al., 2020). Nevertheless, only 3D culture conditions simulate important tumor mircroenvironment characteristics, anti-apoptotic features and finally drug resistance (Imamura et al., 2015; Langhans, 2018). In a study of Imamura *et al.* it has been demonstrated that three breast cancer cell lines cultivated in 3D culture showed greater resistance to paclitaxel and doxorubicin compared to the 2D-cultured cells. One of the underlying molecular mechanisms is the increased expression of cleaved poly (ADP-ribose) polymerase (PARP) in 2D cell cultures compared to 3D. PARPs are a group of enzymes that are responsible for detecting and repairing damaged DNA and play an essential role in cell function and survival. Therefore, a higher expression of PARP enzymes means higher drug sensitivity (Imamura et al., 2015). This provides a further argument for the importance of 3D tumor cell models in applied research.

Here, evaluation of the metabolic activity by XTT revealed the absolute half-maximal inhibitory concentrations in 3D cell culture for the tri-cultures as 50 µM for gefitinib and 50 nM for paclitaxel. A decreased vascularization could be observed after treatment with 50 µM gefitinib and 50 nM paclitaxel in comparison with the untreated control. Additionally, the cell viability decreased significantly after treatment with the half-maximal inhibitory concentrations up to 43% compared to the control. Furthermore, the results revealed that treatment with gefintinib decreases the VEGF secretion over time and in a drug concentration-dependant manner. It is reported, that EGFR tyrosine kinase inhibitors, as gefitinib, decrease VEGF expression by both hypoxia-inducible factor (HIF)-1–independent and HIF-1–dependent mechanisms (Pore et al., 2006). In contrast, VEGF secretion hardly varies in our cancer model when treated with paclitaxel. These findings do not correspond with the microscopy data which showed decreased vascularization. A potential reason could be paclitaxel resistance in our A549 cell line. In ovarian cancer cases, it has been reported that a high percentage of patients have or develop resistance to paclitaxel. The molecular mechanism for this could be transformations of the A-tubulin gene (Ai et al., 2016). Furthermore, in the ongoing development of the model, HIF expression during the cultivation period should also be analysed in order to adjust the hypoxia cultivation conditions where necessary and thereby optimize the model. Additionally, in 2D cell viability and metabolic activity of assays of A549 cells with different paclitaxel concentrations, we could not observe any reduction in viability, while mono-cultures of HUVECs and ASCs showed sensitivity to paclitaxel. Paclitaxel can therefore have an effect on vascularization and reduce it, but no impact on cancer cells and thus the secretion of VEGF was observed. These findings are similar to those previously reported by other research groups. Haase *et al.* showed that paclitaxel treatment resulted in increased apoptosis markers, like Caspase-3, of tumor spheroids and caused microvascular cell death *in vitro* (Haase et al., 2020). Unterleuthner *et al.* demonstrated, that nintedanib, an antiangiogenic lung cancer drug, significantly inhibited vessel sprouting, and branching ability of co-cultures out of HUVECs and fibroblasts (Unterleuthner et al., 2017) (Bray et al., 2017).

In summary, our 3D lung tri-culture cancer model enables qualitative and quantitative analysis of tumor-vascular structures in a fibrin hydrogel. A549 lung cancer cells have successfully been co-cultured with HUVECs and ASCs. The particularity of our model thereby consists in the detailed *in vivo* imitation of the tumor development and the surrounding microenvironment, since tumor angiogenesis is induced by the initial formation of vascular structures and the subsequent contribution of tumor cells. Our model enables the evaluation of the interaction of tumor-specific therapeutics with a tumor-vascularization network and thereby presents a promising opportunity to both supplement and replace animal experiments in cancer research in future. Furthermore, the 3D tumor-models showed significantly increased resistance to the drugs gefitinib and paclitaxel compared to 2D cultures, displaying the high clinical relevance and need of hydrogel based 3D models. The subsequent step in the development of the model is based on the incorporation of patients’ own tumor cells as a patient-specific diagnostic model and drug model.

## Data Availability

The original contributions presented in the study are included in the article/Supplementary Files, further inquiries can be directed to the corresponding author
