# Supplementary material for: Establishment of a Pre-vascularized 3D Lung Cancer Model in Fibrin Gel—Influence of Hypoxia and Cancer-Specific Therapeutics
Source: Front Bioeng Biotechnol. 2021 Oct 14;9:761846. doi: 10.3389/fbioe.2021.761846 (PMC8551668; doi:10.3389/fbioe.2021.761846)
Supplement: Supplementary file 1 [file DataSheet1.docx]

Supplementary Material


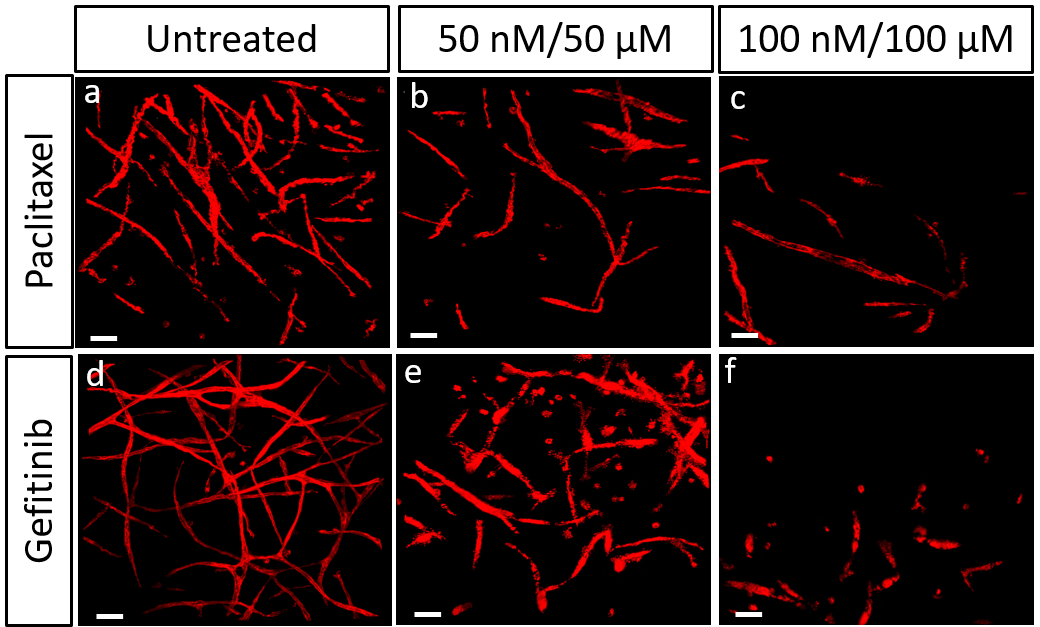


**Supplementary Figure 1. : CD31-stained HUVECs (red) in tri-cultures under hypoxic culture conditions after treatment with 0; 50 and 100 µM gefitinib (a-c, TPLSM images) and 0; 50 and 100 nM paclitaxel for 72 hours (d-f, TPLSM images). Scale bar: 50 µm**


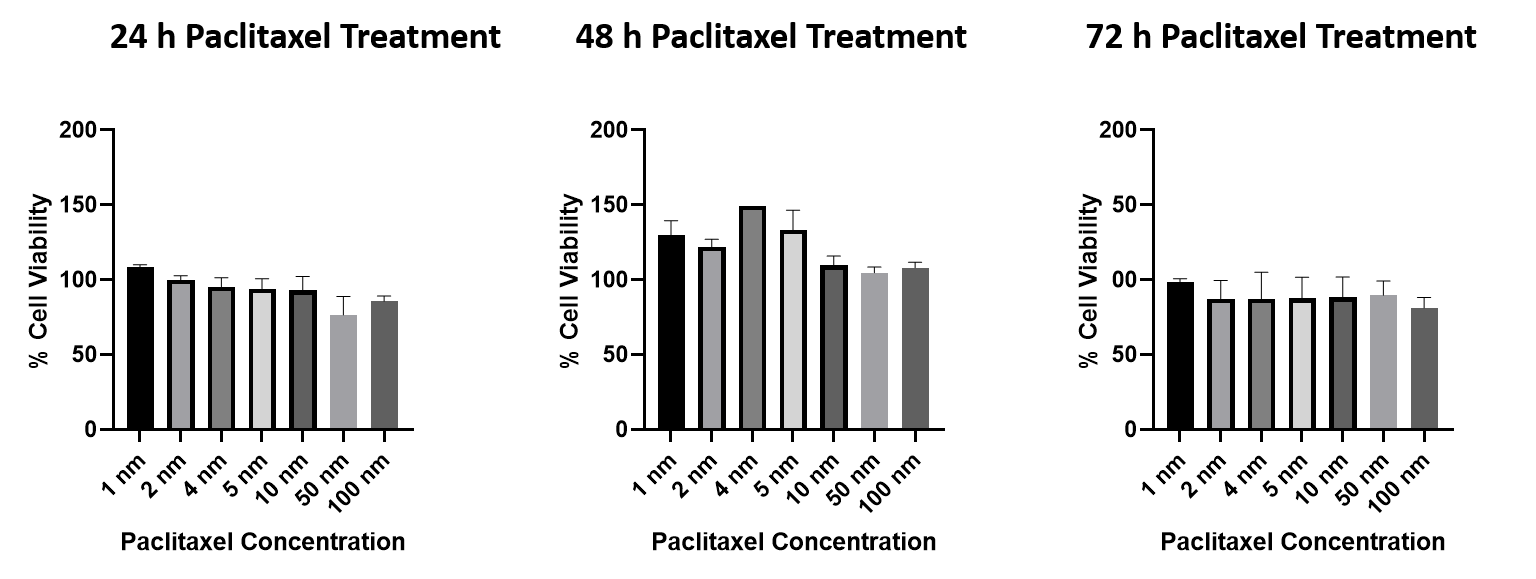


**Supplementary Figure 2. : Cell survival assay of A549 mono-cultures in 2D after treatment with different doses of paclitaxel.**
